# Ocular Point-of-Care Ultrasonography to Diagnose Posterior Chamber Abnormalities: A Systematic Review and Meta-analysis

**DOI:** 10.1001/jamanetworkopen.2019.21460

**Published:** 2020-02-19

**Authors:** Steven L. Propst, Jonathan M. Kirschner, Christian C. Strachan, Steven K. Roumpf, Laura M. Menard, Elisa J. Sarmiento, Benton R. Hunter

**Affiliations:** 1 Department of Emergency Medicine, Indiana University School of Medicine, Indianapolis; 2 Department of Emergency Medicine, CoxHealth, Springfield, Missouri; 3 Ruth Lilly Medical Library, Indiana University School of Medicine, Indianapolis

## Abstract

**Importance:**

Diagnosing posterior chamber ocular abnormalities typically requires specialist assessment. Point-of-care ultrasonography (POCUS) performed by nonspecialists, if accurate, could negate the need for urgent ophthalmologist evaluation.

**Objective:**

This meta-analysis sought to define the diagnostic test characteristics of emergency practitioner–performed ocular POCUS to diagnose multiple posterior chamber abnormalities in adults.

**Data Sources:**

PubMed (OVID), MEDLINE, EMBASE, Cochrane, CINAHL, and SCOPUS were searched from inception through June 2019 without restrictions. Conference abstracts and trial registries were also searched. Bibliographies of included studies and relevant reviews were manually searched, and experts in the field were queried.

**Study Selection:**

Included studies compared ocular POCUS performed by emergency practitioners with a reference standard of ophthalmologist evaluation. Pediatric studies were excluded. All 116 studies identified during abstract screening as potentially relevant underwent full-text review by multiple authors, and 9 studies were included.

**Data Extraction and Synthesis:**

In accordance with PRISMA guidelines, multiple authors extracted data from included studies. Results were meta-analyzed for each diagnosis using a bivariate random-effects model. Data analysis was performed in July 2019.

**Main Outcomes and Measures:**

The outcomes of interest were diagnostic test characteristics of ocular POCUS for the following diagnoses: retinal detachment, vitreous hemorrhage, vitreous detachment, intraocular foreign body, globe rupture, and lens dislocation.

**Results:**

Nine studies (1189 eyes) were included. All studies evaluated retinal detachment, but up to 5 studies assessed each of the other diagnoses of interest. For retinal detachment, sensitivity was 0.94 (95% CI, 0.88-0.97) and specificity was 0.94 (95% CI, 0.85-0.98). Sensitivity and specificity were 0.90 (95% CI, 0.65-0.98) and 0.92 (95% CI, 0.75-0.98), respectively, for vitreous hemorrhage and were 0.67 (95% CI, 0.51-0.81) and 0.89 (95% CI, 0.53-0.98), respectively, for vitreous detachment. Sensitivity and specificity were high for lens dislocation (0.97 [95% CI, 0.83-0.99] and 0.99 [95% CI, 0.97-1.00]), intraocular foreign body (1.00 [95% CI, 0.81-1.00] and 0.99 [95% CI, 0.99-1.00]), and globe rupture (1.00 [95% CI, 0.63-1.00] and 0.99 [95% CI, 0.99-1.00]). Results were generally unchanged in sensitivity analyses of studies with low risk of bias.

**Conclusions and Relevance:**

This study suggests that emergency practitioner–performed ocular POCUS is an accurate test to assess for retinal detachment in adults. Its utility in diagnosing other posterior chamber abnormalities is promising but needs further study.

## Key Points

**Question:**

What is the accuracy of ocular point-of-care ultrasonography performed by emergency practitioners in diagnosing posterior chamber abnormalities?

**Findings:**

This systematic review and meta-analysis found high sensitivity and specificity for diagnosing retinal detachment across 9 studies (1189 eyes). Accuracy for other abnormalities was less well studied but appeared to be high for lens dislocation, intraocular foreign body, and globe rupture and moderately to poorly accurate for vitreous hemorrhage and vitreous detachment.

**Meaning:**

Emergency practitioner–performed ultrasonography has high accuracy for retinal detachment, and although its use for other posterior chamber abnormalities appears promising, it requires further study.

## Teaser

This meta-analysis examines the accuracy of point-of-care ultrasonography performed by emergency practitioners in diagnosing posterior chamber ocular abnormalities.

## Introduction

Ophthalmologic emergencies account for approximately 1.3 million emergency department (ED) visits in the United States annually.^1^ Many such cases are secondary to trauma, and more than 40% of ED ocular conditions are emergent.^2,3^ Diagnoses involving the external eye and anterior chamber can typically be made on the basis of history and physical examination alone. However, visualizing and diagnosing posterior chamber abnormalities can be more challenging for nonspecialists because of inexperience, limitations of available equipment, and less-than-ideal examination conditions. Most physicians agree that fundoscopy is an important physical examination skill; however, many do not perform the examination when indicated or lack confidence in their ability to perform it effectively.^4,5^

Several studies have demonstrated that emergency practitioner (EP)–performed ocular point-of-care ultrasonography (POCUS) can be used to diagnose retinal detachment, as well as other ocular emergencies such as vitreous hemorrhage or detachment, lens dislocation, intraocular foreign bodies, or globe rupture.^6,7,8,9,10,11,12,13^ The use of POCUS to diagnose retinal detachment has garnered attention because of the time sensitivity of treating the detachment and the difficulty for nonspecialists in making the diagnosis with physical examination and fundoscopy.

A recently published systematic review and meta-analysis^14^ demonstrated sensitivity and specificity of 94% and 96%, respectively, for ocular POCUS to diagnose retinal detachment. The meta-analysis^14^ included studies of POCUS performed by a range of practitioners, including radiologists and ophthalmologists, and included a total of 844 patients from 11 studies. Subsequently, 2 large, prospective studies^15,16^ have added substantially to the existing data regarding EP-performed ocular POCUS. Lahham and colleagues^15^ (225 patients) and Ojaghihaghighi et al^16^ (232 patients) studied ocular POCUS to diagnose multiple posterior chamber abnormalities. Because of the expanding evidence regarding EP-performed POCUS for the evaluation of retinal detachment,^6,7,11,12,13,15,16,17,18^ and because, to our knowledge, no previous systematic review has evaluated the diagnostic utility of POCUS in diagnosing other posterior chamber abnormalities, the present systematic review and meta-analysis was undertaken to further define the test characteristics of EP-performed ocular POCUS in diagnosing posterior chamber abnormalities.

## Methods

This study was exempt from institutional review board approval because no patient data were used, in accordance with 45 CFR §46.102(f). This systematic review follows the Preferred Reporting Items for Systematic Reviews and Meta-analyses (PRISMA) reporting guideline for performing and reporting systematic reviews. It has been registered on PROSPERO.

### Study Eligibility Criteria

Studies assessing the diagnostic accuracy of POCUS to diagnose posterior chamber abnormalities in adults were included if they met the following 3 criteria: POCUS examinations were performed by EPs, the diagnostic reference standard included formal ophthalmologic examination (examination and diagnosis by an ophthalmologist), and sufficient information was provided to create a 2 × 2 table for the test characteristics to diagnose at least 1 of our predefined posterior chamber abnormalities (retinal detachment, vitreous hemorrhage, vitreous detachment, lens dislocation, intraocular foreign body, and ruptured or open globe). Exclusion criteria were studies of patients younger than 18 years, POCUS operators who were not EPs, and case reports or case series of fewer than 10 patients.

### Search Strategy

A medical librarian (L.M.M.) conducted a systematic search based on PRISMA guidelines. PubMed, (OVID) MEDLINE, EMBASE, Cochrane, CINAHL, and SCOPUS were searched to identify studies published between January 1, 1960, and June 1, 2019. A combination of Medical Subject Headings terms and keywords pertaining to the problem or population, intervention, and setting were identified in collaboration between the senior author (B.R.H.) and the librarian conducting the search. We queried experts in the field and manually searched the bibliographies of included studies and relevant reviews for additional studies. In addition, a supplemental search of the gray literature was performed, including conference abstracts and clinical trial registries (eg, ClinicalTrials.gov). The complete search strategy (PubMed) is available in the eAppendix in the Supplement.

After the removal of duplicates, all titles and abstracts identified by the search were screened independently by 2 authors (C.C.S. and S.K.R.). Full text was obtained for all articles deemed possibly relevant by either screener. Full-text reviews were performed independently by 2 authors (S.L.P. and B.R.H.) to determine final eligibility for inclusion in the review. Disagreements about inclusion were to be resolved through discussion, with adjudication by a third author (J.M.K.) if necessary.

### Quality Appraisals

The quality of each individual study was appraised using the Quality Appraisal of Diagnostic Accuracy Studies–2 instrument. Briefly, the instrument allows for assessment of the risk of bias in each of 4 domains: patient selection, performance of the index test, performance of the reference standard, and patient flow and timing. The instrument was applied to each study by 2 authors independently (S.L.P. and J.M.K.), with disagreements resolved through discussion and adjudication by a third author (B.R.H.) if necessary.

### Data Abstraction

In pairs of 2, 4 authors (S.L.P., C.C.S, S.K.R., and B.R.H.) independently abstracted data from each individual study using structured forms. Data abstraction included the year of publication, setting (including country and type of ED), study design, inclusion and exclusion criteria, and true positives, false positives, true negatives, and false negatives for each predefined diagnosis that was reported in the study. Any discrepancies were resolved through discussion, with adjudication by a third author (B.R.H. or J.M.K) if necessary.

### Outcomes

The outcomes of interest were diagnostic test characteristics (sensitivity, specificity, and positive and negative likelihood ratios) of ultrasonography for each of the following diagnoses: retinal detachment, vitreous hemorrhage, vitreous detachment, lens dislocation, intraocular foreign body, and globe rupture. Preplanned sensitivity analyses were the same outcomes among studies: at low risk of bias and with highly experienced (fellowship trained or ultrasonography directors) vs minimally trained ultrasonographers.

### Statistical Analysis

Study results were meta-analyzed using a random-effects model to generate summary estimates of sensitivity, specificity, and negative and positive likelihood ratios with 95% CIs. For analyses of at least 4 studies, a bivariate model was used in the MIDAS module of StataMP statistical software version 13 (StataCorp). For analyses combining 3 or fewer studies, a univariate model in the DIAGT Stata module was used. The *I*^2^ statistic is reported as a quantification of the proportion of total variability due to heterogeneity for all analyses of at least 3 studies. *I*^2^ values of 25%, 50%, and 75% generally denote a small, moderate, and high proportion of variability, respectively, due to heterogeneity. Results were combined regardless of *I*^2^ values. Forest plots for sensitivity and specificity were created with RevMan statistical software version 5.3 (The Nordic Cochrane Center). Data analysis was performed in July 2019.

## Results

### Search Results

**Figure 1.**
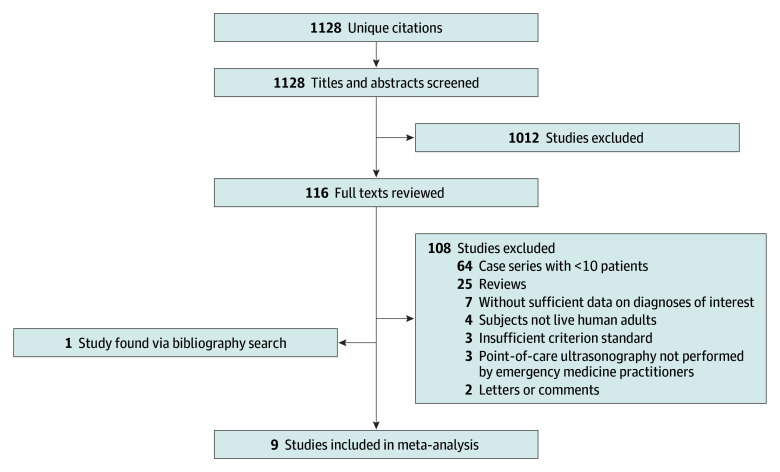
Flow Diagram of Study Identification Flowchart shows study inclusion and exclusion criteria.

Figure 1 outlines the flow of study identification. The initial search returned 1128 unique citations. After screening of titles and abstracts, 116 articles underwent full-text review, of which 8 met the inclusion and exclusion criteria. One additional study was identified by the bibliography search, resulting in 9 studies eligible for meta-analysis. There were no disagreements regarding inclusion and exclusion of studies after full text review.

### Included Studies

Characteristics of the 9 included studies are outlined in Table 1. Five were performed in the United States,^6,11,12,15,17^ 2 in Canada, and 1 each in China and Iran.^7,13,16,18^ Most studies were performed in urban academic centers. Patient enrollment ranged from 61 to 232 patients. Some studies allowed for bilateral ocular POCUS, resulting in more data points (1189 eyes) than total patients enrolled (1083 patients). All included studies evaluated POCUS to diagnose retinal detachment, and 5 studies also evaluated POCUS in the diagnosis of other abnormalities. There were 8 prospective cohort studies,^6,7,11,12,13,15,16,18^ and 1 retrospective study.^17^ All studies used a linear ultrasonography probe. Seven of the studies^6,7,11,12,15,17,18^ used either a 7.5-MHz or 10-MHz frequency probe. One study^13^ used a range of frequencies from 6 MHz to 13 MHz, and another study^16^ used a frequency range of 7 MHz to 15 MHz. Emergency medicine faculty and residents appeared to perform POCUS examinations in all studies. One study^7^ also included emergency medical students, and 2 studies^12,15^ included physicians’ assistants. Six studies^7,13,15,16,17,18^ had been published since 2016. One study^16^ exclusively evaluated ocular trauma, and another study^15^ excluded ocular trauma. The remaining studies either included a mix of patients with and without trauma or did not specify whether traumatically injured patients were included.

**Table 1.** Characteristics of Included Studies

Study	Setting	Country	Ultrasonographers	Patient Inclusions	Patients, No. (Total No. of Eyes)	Patient Exclusions
Blaivas et al,^6^ 2002	Suburban community ED	United States	3 Attending and 5 resident physicians	Ocular trauma or vision changes	49	Binocular vision loss or no confirmatory testing
Chu et al,^18^ 2017	Large urban ED	China	6 Emergency physician volunteers with previous experience	Adults with <48 h of visual symptoms	139	Preexisting detachment; hemodynamically unstable; suspected globe rupture
Jacobsen et al,^17^ 2016	Urban academic ED	United States	26 Attending and 30 resident physicians; minimal training required	Concern for RD; billed for ocular point-of-care ultrasonography	109	Concern for globe rupture; no ophthalmology consultation
Kim et al,^13^ 2019	Urban academic ED	Canada	20 Staff, 2 fellows, and 8 resident physicians; minimal training required	Acute flashers or floaters	115	Symptoms lasting >7 d; known prior RD; advanced cataract; ocular surgery in last 2 wk
Lahham et al,^15^ 2019	Academic and county EDs	United States	75 Total attending and resident physicians and physicians’ assistants	Concern for RD, vitreous hemorrhage, or vitreous detachment and undergoing ophthalmology consultation	225	Patients aged <18 y; non–English speaking; ocular trauma; suspected globe rupture
Ojaghihaghighi et al,^16^ 2019	Academic ED	Iran	2 Emergency physicians with extensive training	Facial trauma warranting diagnostic evaluation	232 (351)	Unable to consent; unable to undergo computed tomography or ophthalmology examination; suspected globe rupture
Shinar et al,^12^ 2011	Large urban teaching ED	United States	1 Attending and 27 resident physicians and 3 physicians’ assistants	Concern for RD	90 (92)	Unable to obtain ophthalmology consultation
Woo et al,^7^ 2016	Urgent ophthalmology clinic	Canada	Emergency medicine attending and resident physicians and medical students	Patient aged >18 y; referred to ophthalmology clinic with <7 d of flashers, floaters, or field deficits	62	Previous RD or ocular surgery; suspected globe rupture; anterior chamber abnormality
Yoonessi et al,^11^ 2010	Academic ED	United States	2 Attending and 13 resident physicians	Visual changes; getting an ophthalmology consultation for RD; ultrasonography could be performed before consultation	48	Unable to consent; non–English speaking; preestablished diagnosis

Abbreviations: ED, emergency department; RD, retinal detachment.

### Risk of Bias

The Quality Appraisal of Diagnostic Accuracy Studies–2 assessments for quality of the included studies are presented in Table 2. The included studies ranged from low to high overall risk of bias. Eight of 9 studies were at unclear risk of selection bias because most used convenience sampling. The applicability of the index test was unclear in several studies because the operators were either extensively trained in POCUS or the experience level of ultrasonography operators and their relative contribution to the study result was unclear. Our inclusion criteria dictated that all studies used formal ophthalmologic evaluation as the reference standard; however, in 3 of 9 studies,^6,12,17^ the ophthalmologist was not necessarily masked to the POCUS result. Blaivas et al^6^ allowed for a reference standard of either CT scan or formal ophthalmologist evaluation, but only those patients for whom the final diagnosis was based on ophthalmologist examination were included in the quantitative analysis. Several studies had lost patients and delayed and differential reference standard assessment.

**Table 2.** Quality Appraisal of Diagnostic Accuracy Studies–2 Critical Appraisal Ratings

Study	Patient Selection		Index Tests		Reference Standard		Flow and Timing
	Risk of Bias	Applicability	Risk of Bias	Applicability	Risk of Bias	Applicability	Risk of Bias
Woo et al,^7^ 2016	Unclear	Low	Low	Low	Low	Low	Low
Yoonessi et al,^11^ 2010	Unclear	Low	Low	Low	Low	Low	Low
Blaivas et al,^6^ 2002	Unclear	Low	Low	Unclear	Unclear	Low	Unclear
Ojaghihaghighi et al,^16^ 2019	Low	Low	Low	High	Low	Low	Low
Shinar et al,^12^ 2011	Unclear	Low	Low	Low	High	Low	Unclear
Chu et al,^18^ 2017	Unclear	Low	Low	Unclear	Low	Low	Low
Jacobsen et al,^17^ 2016	Unclear	Low	High	High	Unclear	Low	Low
Kim et al,^13^ 2019	Unclear	Low	Low	Low	Low	Low	Unclear
Lahham et al,^15^ 2019	Unclear	Low	Low	Low	Low	Low	Unclear

### Main Results

**Figure 2.**
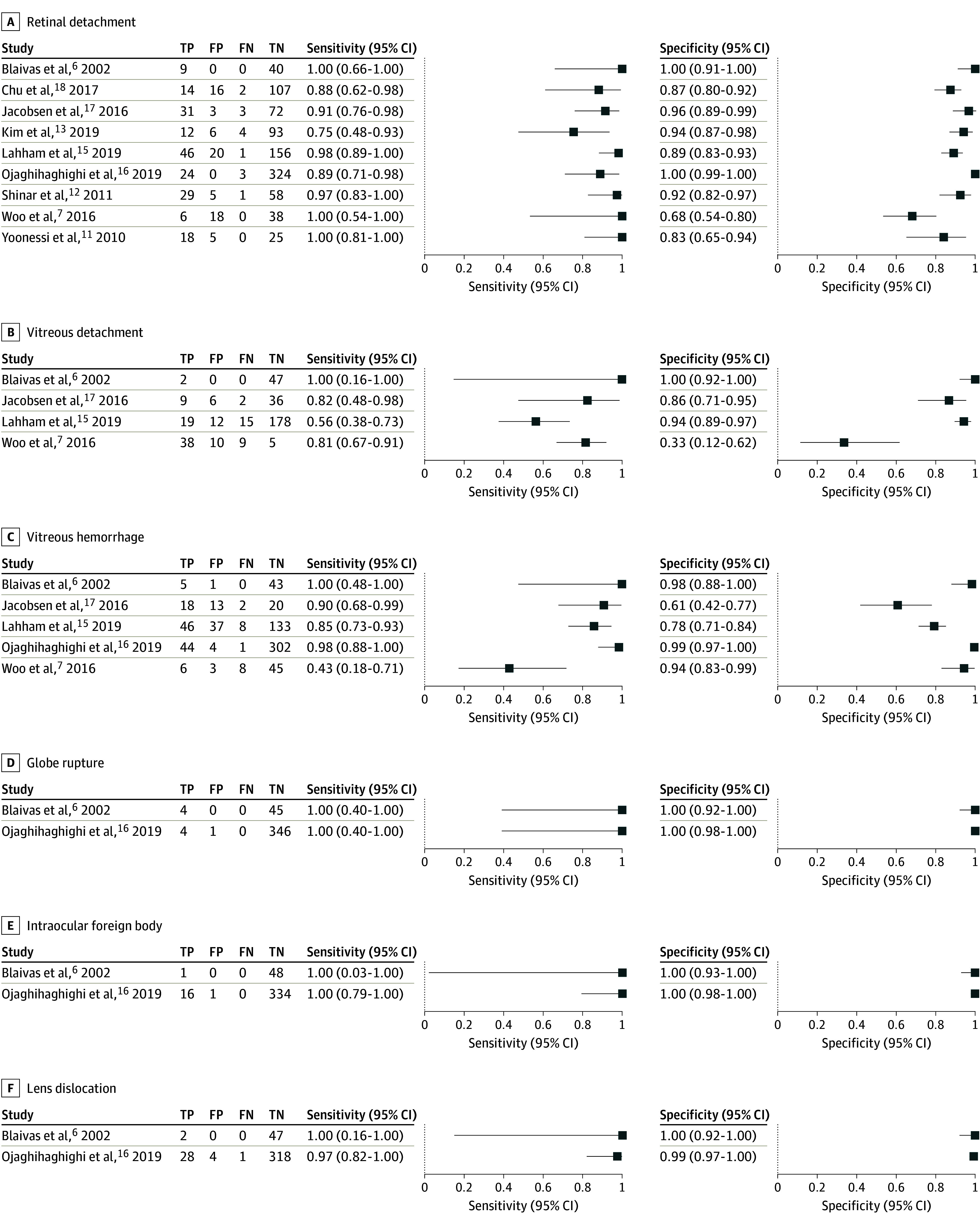
Forest Plots of Primary Analyses Plots show the sensitivity and specificity of point-of-care ultrasonography performed by emergency practitioners for 6 ocular conditions for the studies included in the meta-analysis. FN indicates false negative; FP, false positive; TN, true negative; and TP, true positive.

Results of the meta-analyses are outlined in Table 3, and forest plots of individual study results are provided in Figure 2. Among the 9 studies,^6,7,11,12,13,15,16,17,18^ the combined sensitivity for POCUS for retinal detachment was 0.94 (95% CI, 0.88-0.97; *I*^2^ = 54%) and specificity was 0.94 (95% CI, 0.85-0.98; *I*^2^ = 92%). Positive and negative likelihood ratios were 16.6 (6.1-45.3) and 0.064 (0.031-0.130), respectively. The overall prevalence of retinal detachment in the included studies was 26%. The diagnosis of vitreous hemorrhage was evaluated in 5 studies,^6,7,15,16,17^ demonstrating a sensitivity of 0.90 (95% CI, 0.65-0.98; *I*^2^ = 92%) and specificity of 0.92 (95% CI, 0.75-0.98; *I*^2^ = 96%). Positive and negative likelihood ratios were 11.7 (95% CI, 3.1-44.3) and 0.112 (95% CI, 0.027-0.459), respectively. Vitreous detachment was evaluated by POCUS in 4 studies,^6,7,15,17^ with a combined sensitivity of 0.67 (95% CI, 0.51-0.81; *I*^2^ = 75%) and specificity of 0.89 (95% CI, 0.53-0.98; *I*^2^ = 96%). Positive and negative likelihood ratios were 6.2 (95% CI, 1.2-31.3) and 0.36 (95% CI, 0.25-0.53), respectively. Lens dislocation, intraocular foreign body, and globe rupture were each evaluated by the same 2 studies,^6,16^ demonstrating sensitivities of 0.97 (95% CI, 0.83-0.99), 1.00 (95% CI, 0.81-1.00), and 1.00 (95% CI, 0.63-1.00), respectively. Specificities for lens dislocation, intraocular foreign body, and globe rupture were 0.99 (95% CI, 0.97-1.00), 0.99 (95% CI, 0.99-1.00), and 0.99 (95% CI, 0.99-1.00), respectively.

**Table 3.** Results of Meta-analyses

Diagnosis	Studies, No.	Eyes, No.	Sensitivity (95% CI)	*I*^2^, %	Specificity (95% CI)	*I*^2^, %	Positive Likelihood Ratio (95% CI)	Negative Likelihood Ratio (95% CI)
Retinal detachment	9	1189	0.94 (0.88-0.97)	54	0.94 (0.85-0.98)	92	16.6 (6.1-45.3)	0.064 (0.031-0.130)
Vitreous hemorrhage	5	739	0.90 (0.65-0.98)	92	0.92 (0.75-0.98)	96	11.7 (3.1-44.3)	0.112 (0.027-0.459)
Vitreous detachment	4	388	0.67 (0.51-0.81)	75	0.89 (0.53-0.98)	96	6.2 (1.2-31.3)	0.36 (0.25-0.53)
Lens dislocation	2	400	0.97 (0.83-0.99)		0.99 (0.97-1.00)		89.3 (33.0-237.0)	0.03 (0.00-0.22)
Intraocular foreign body	2	400	1.00 (0.81-1.00)		0.99 (0.99-1.00)		383.0 (54.0-2712.0)	0
Globe rupture	2	400	1.00 (0.63-1.00)		0.99 (0.99-1.00)		392.0 (55.0-2779.0)	0
Sensitivity analyses of studies with low risk of bias								
Retinal detachment	6	940	0.94 (0.83-0.98)	60	0.93 (0.76-0.98)	94	11.6 (9.2-14.8)	0.07 (0.04-0.13)
Vitreous detachment	2	286	0.70 (0.59-0.80)		0.89 (0.84-0.93)		6.60 (4.31-9.97)	0.330 (0.236-0.466)
Vitreous hemorrhage	3	637	0.85 (0.77-0.91)	92	0.92 (0.89-0.94)	60	10.10 (7.55-13.60)	0.160 (0.106-0.255)

### Sensitivity Analyses

Sensitivity analysis of studies based on ultrasonographer experience was not possible because of the lack of clarity about the experience of POCUS operators in most studies. Combining results from only the 6 studies^7,11,13,15,16,18^ with low risk of bias evaluating retinal detachment did not substantially change the estimate of accuracy, with combined sensitivity and specificity of 0.94 (95% CI, 0.83-0.98) and 0.93 (95% CI, 0.76-0.98), respectively. Analyzing the 3 studies^7,15,16^ with low risk of bias for the diagnosis of vitreous hemorrhage decreased the sensitivity slightly to 0.85 (95% CI, 0.77-0.91) and did not affect specificity (0.92; 95% CI, 0.89-0.94). Only 2 studies^7,15^ were included in the low risk of bias analysis for vitreous detachment, yielding results similar to those for the primary analysis, with sensitivity of 0.70 (95% CI, 0.59-0.80) and specificity of 0.89 (95% CI, 0.84-0.93). Results of the low risk of bias sensitivity analyses are outlined in Table 3. Low risk of bias sensitivity analyses were not performed for lens dislocation, intraocular foreign body, or globe rupture, because only 1 study^16^ would have been included.

## Discussion

To our knowledge, this is the first systematic review evaluating EP-performed ocular POCUS for a wide range of posterior chamber diagnoses; we found generally high diagnostic accuracy across the 6 diagnoses assessed and exceptionally high accuracy in diagnosing retinal detachment. We chose to include only studies of EP-performed POCUS because radiologist-performed POCUS is not widely available and the presence of an ophthalmologist to perform POCUS likely negates the need to perform the test. This resulted in the exclusion of 5 studies of radiologist-performed POCUS included in the systematic review by Gottlieb and colleagues,^14^ which included a wide range of POCUS operators assessing for retinal detachment. Nonetheless, our results were consistent with those reported by Gottlieb et al.^14^ In addition, we found good-to-excellent diagnostic accuracy in assessing for vitreous hemorrhage, lens dislocation, intraocular foreign body, and globe rupture, although the 95% CIs around some of our results are wide. Interestingly, POCUS appears to be less accurate in diagnosing vitreous detachment.

Confidence in the estimate of diagnostic accuracy for retinal detachment is bolstered by narrow 95% CIs resulting from the inclusion of several studies and the fact that the results were essentially unchanged when only studies with low risk of bias were included. Retinal detachment must be considered in the differential diagnosis of acute visual conditions because of the time sensitivity of the diagnosis. Retinal detachment is uncommon, with an incidence of about 10 cases per 100 000 population per year.^19^ Rhegmatogenous retinal detachment is the most common type of retinal detachment, and it occurs when a tear or break in the retina allows vitreous fluid to flow into the subretinal space, causing separation of the neurosensory retina. Symptoms often consist of flashing lights, floaters, or loss of central or peripheral vision. The time sensitivity is associated with the potential for further invasion of vitreous fluid into the subretinal space, lifting the retina, and leading to detachment of the macula and irreversible vision loss.^20,21^ Our results should give EPs confidence that negative POCUS findings substantially decrease the pretest probability of retinal detachment and can be used to minimize the potential for a missed retinal detachment. It should be noted that POCUS for retinal tears has poor sensitivity (47.8%),^7^ which is on the same spectrum as that for retinal detachment; however, a tear or hole may occur before the neurosensory retina substantially lifts away or detaches from the retinal pigment epithelium.^22^ As a result, timely ophthalmology follow-up should be encouraged if POCUS demonstrates no retinal detachment, because the prevalence of retinal tear is 14% among patients with acute onset of floaters or flashes.^23^

The overall prevalence of retinal detachment in the included studies was 26%, which is slightly higher than the previously reported ED-to-ophthalmology referral incidence of approximately 10% to 15%.^24,25^ Applying a negative likelihood ratio of 0.064 to a pretest probability of 26% results in a posttest probability of 2%. For patients with even lower pretest probability, these results may give EPs the confidence to effectively exclude retinal detachment after negative ocular POCUS findings, or at least to consider allowing for expedient ophthalmologic follow-up rather than insisting on ophthalmologic evaluation before the patient leaves the ED.

Ocular POCUS was also accurate in diagnosing vitreous hemorrhage, which is common and is typically due to either rupture of normal vessels via mechanical forces or hemorrhage from pathologic blood vessels, such as neovascularization in diabetic retinopathy, leading to bleeding and clot formation in the vitreous. The blood is cleared slowly at a rate of 1% per day.^26^ Although vitreous hemorrhage may be associated with retinal tears, absent that complication, it is not a time-sensitive emergency, and patients can follow up nonemergently to make sure the blood is clearing appropriately and to treat the underlying risk factors.^26^ Vitrectomy is often reserved for nonclearing vitreous hemorrhage.^21,27^ Interestingly, EP-performed POCUS was poor at diagnosing vitreous detachment, a condition that occurs when the posterior vitreous separates from the retina. This disease process is also nonemergent, absent any associated retinal tear. There are no recommended therapies, and surgery is not required in uncomplicated cases. The patient should follow up for monitoring of disease progression because there is a 3.4% chance of developing a retinal tear within 6 weeks.^21,23^

Because of the limited number of studies evaluating vitreous hemorrhage and vitreous detachment and the resultant small sample sizes, the 95% CIs around these estimates are wide, and POCUS cannot be strongly recommended to confirm or exclude these diagnoses, especially vitreous detachment, where the point estimate for sensitivity was low (0.67). In addition, given that these diagnoses can be associated with retinal detachments, substantial clinical suspicion for either diagnosis should not be negated by a POCUS examination with negative findings, and timely, but not emergent, ophthalmology follow-up is warranted.

Because of the small number of studies that assessed the diagnostic accuracy of POCUS for lens dislocation, intraocular foreign body, and globe rupture and the very small number of true positives, confidence in these results is very low. In fact, many studies explicitly excluded patients for whom there was concern for globe rupture for fear of applying any pressure to a potentially open globe. Although neither study^6,16^ that assessed for globe rupture reported adverse events, the safety of ocular POCUS in this setting has not been proven and cannot be recommended. Similarly, assessment for intraocular foreign body implies concern for a penetrating injury, a condition where pressure on the eye itself could potentially be dangerous.

### Limitations

There are several limitations to consider when interpreting our results, mainly concerning the number and quality of included studies. There were a small number of studies evaluating POCUS to diagnose abnormalities other than retinal detachment. Most notably, only 2 studies^6,16^ evaluated the clinical outcomes of lens dislocation, intraocular foreign body, and globe rupture. These diagnoses were uncommon, leading to few true positives and, consequently, large 95% CIs around the estimates for sensitivity. The 95% CIs around the estimates of sensitivity and specificity for retinal detachment were substantially narrower. The incidence of retinal detachment in our meta-analysis was 26%, which is higher than in previous studies,^24,25^ which found prevalences of 11% and 15%. If spectrum bias exists, then the sensitivity could be lower in lower-risk patients.

Three of the 9 included studies^6,12,17^ were at high risk of bias. However, the subanalyses of only studies with low risk of bias produced similar results across diagnoses, suggesting that risk of bias may have had a minimal effect on the estimates. Although 1 of the 2 studies^6,16^ reporting diagnostic accuracy for lens dislocation, intraocular foreign body, and globe rupture was high risk of bias, the study with low risk of bias by Ojaghihaghighi and colleagues^16^ was much larger and dominated the results of those meta-analyses.

We attempted to minimize clinical heterogeneity by including only studies of adult patients for whom POCUS was performed by EPs and in which the reference standard was ophthalmologic examination. Despite this, statistical heterogeneity was moderate to high across all analyses including more than 2 studies. This may be due to differences in proficiency of operators and ultrasonography experience, but there were insufficient data about individual operator experience to test this hypothesis in a sensitivity analysis. Point-of-care ultrasonography is now a required skill for emergency medicine residents,^28^ and many EPs have experience with its use. However, the applicability of POCUS as a diagnostic tool will be affected by the ultrasonography operator’s experience and skill level, which is still quite variable among EPs. Operator experience in the included studies ranged from medical students with presumably minimal experience and training to ultrasonography fellowship directors and attending physicians with extensive experience. Most of the included studies allowed for a wide range of operators, with minimal requirements for previous training. Several studies allowed physicians to enroll patients after 1 to 2 hours of lecture or training on ocular ultrasonography, with some including a hands-on model demonstration.

We think that there is a low risk of applicability bias overall, because requirements for training were minimal. However, it should be noted that most studies included operators who had volunteered to participate, and it is likely that those with ultrasonography interest or experience were more likely to participate in these studies than others. In addition, the largest overall study^16^ included only 2 operators who had extensive training in ocular ultrasonography.

## Conclusions

Trained EPs perform ocular POCUS with high enough sensitivity and specificity to rule out retinal detachment in low-risk patients and to rule in the diagnosis in those at high risk. Limited data suggest that EP-performed POCUS is moderately accurate for diagnosing vitreous hemorrhage and poorly accurate for diagnosing vitreous detachment. Preliminary data suggest that EP-performed POCUS is highly accurate to diagnose lens dislocation, intraocular foreign body, and globe rupture, but further evidence is required before confidently recommending its routine use for these indications.

## Notes

Supplement. **eAppendix.** Complete Search Strategy (PubMed)
