# Supplementary material for: Ocular Point-of-Care Ultrasonography to Diagnose Posterior Chamber Abnormalities: A Systematic Review and Meta-analysis
Source: JAMA Netw Open. 2020 Feb 19;3(2):e1921460. doi: 10.1001/jamanetworkopen.2019.21460 (PMC12527417; doi:10.1001/jamanetworkopen.2019.21460)
Supplement: Supplement. — eAppendix. Complete Search Strategy (PubMed) [file jamanetwopen-e1921460-s001.pdf]

Supplementary Online Content

Propst SL, Kirschner JM, Strachan CC, et al. Ocular point-of-care ultrasonography to diagnose posterior chamber abnormalities: a systematic review and meta-analysis. *JAMA Netw Open*. 2020;3(2):e1921460. doi:10.1001/jamanetworkopen.2019.21460

eAppendix. Complete Search Strategy (PubMed)

This supplementary material has been provided by the authors to give readers additional information about their work.

© 2020 Propst SL et al. *JAMA Network Open.*

eAppendix. Complete Search Strategy (PubMed)

#1
Search: ((((("emergency medical services"[MeSH Terms] OR ("emergency"[All Fields] AND "medical"[All Fields] AND "services"[All Fields]) OR "emergency medical services"[All Fields] OR ("emergency"[All Fields] AND "service"[All Fields]) OR "emergency service"[All Fields]) OR "Emergency Service, Hospital"[Mesh]) OR ("emergency medicine"[MeSH Terms] OR ("emergency"[All Fields] AND "medicine"[All Fields]) OR "emergency medicine"[All Fields])) OR ("emergency service, hospital"[MeSH Terms] OR ("emergency"[All Fields] AND "service"[All Fields] AND "hospital"[All Fields]) OR "hospital emergency service"[All Fields] OR ("emergency"[All Fields] AND "department"[All Fields]) OR "emergency department"[All Fields]))
317040 Results

#2
Search: (((((("diagnostic imaging"[Subheading] OR ("diagnostic"[All Fields] AND "imaging"[All Fields]) OR "diagnostic imaging"[All Fields] OR "diagnostic imaging"[MeSH Terms] OR ("diagnostic"[All Fields] AND "imaging"[All Fields])) OR (bedside[All Fields] AND ("diagnostic imaging"[Subheading] OR ("diagnostic"[All Fields] AND "imaging"[All Fields]) OR "diagnostic imaging"[All Fields] OR "ultrasound"[All Fields] OR "ultrasonography"[MeSH Terms] OR "ultrasonography"[All Fields] OR "ultrasound"[All Fields] OR "ultrasonics"[MeSH Terms] OR "ultrasonics"[All Fields]))) OR POCUS[All Fields]) OR (("point-of-care systems"[MeSH Terms] OR ("point-of-care"[All Fields] AND "systems"[All Fields]) OR "point-of-care systems"[All Fields] OR ("point"[All Fields] AND "care"[All Fields]) OR "point of care"[All Fields]) AND ("diagnostic imaging"[Subheading] OR ("diagnostic"[All Fields] AND "imaging"[All Fields]) OR "diagnostic imaging"[All Fields] OR "ultrasound"[All Fields] OR "ultrasonography"[MeSH Terms] OR "ultrasonography"[All Fields] OR "ultrasound"[All Fields] OR "ultrasonics"[MeSH Terms] OR "ultrasonics"[All Fields]))) OR ("diagnostic imaging"[Subheading] OR ("diagnostic"[All Fields] AND "imaging"[All Fields]) OR "diagnostic imaging"[All Fields] OR "ultrasonography"[All Fields] OR "ultrasonography"[MeSH Terms])) OR ("diagnostic imaging"[Subheading] OR ("diagnostic"[All Fields] AND "imaging"[All Fields]) OR "diagnostic imaging"[All Fields] OR "ultrasound"[All Fields] OR "ultrasonography"[MeSH Terms] OR "ultrasonography"[All Fields] OR "ultrasound"[All Fields] OR "ultrasonics"[MeSH Terms] OR "ultrasonics"[All Fields])))
2745210 Results

#3
Search: ((((((((((((("eye foreign bodies"[MeSH Terms] OR ("eye"[All Fields] AND "foreign"[All Fields] AND "bodies"[All Fields]) OR "eye foreign bodies"[All Fields] OR ("eye"[All Fields] AND "foreign"[All Fields] AND "body"[All Fields]) OR "eye foreign body"[All Fields]) OR ("vitreous haemorrhage"[All Fields] OR "vitreous hemorrhage"[MeSH Terms] OR ("vitreous"[All Fields] AND "hemorrhage"[All Fields]) OR "vitreous hemorrhage"[All Fields])) OR ("vitreous detachment"[MeSH Terms] OR ("vitreous"[All Fields] AND "detachment"[All Fields]) OR

© 2020 Propst SL et al. *JAMA Network Open.*

"vitreous detachment"[All Fields])) OR ("vitreous body"[MeSH Terms] OR ("vitreous"[All Fields] AND "body"[All Fields]) OR "vitreous body"[All Fields])) OR ("retrobulbar hemorrhage"[MeSH Terms] OR ("retrobulbar"[All Fields] AND "hemorrhage"[All Fields]) OR "retrobulbar hemorrhage"[All Fields] OR ("retrobulbar"[All Fields] AND "hematoma"[All Fields]) OR "retrobulbar hematoma"[All Fields])) OR ("retinal haemorrhage"[All Fields] OR "retinal hemorrhage"[MeSH Terms] OR ("retinal"[All Fields] AND "hemorrhage"[All Fields]) OR "retinal hemorrhage"[All Fields])) OR ("retinal detachment"[MeSH Terms] OR ("retinal"[All Fields] AND "detachment"[All Fields]) OR "retinal detachment"[All Fields])) OR ("eye infections"[MeSH Terms] OR ("eye"[All Fields] AND "infections"[All Fields]) OR "eye infections"[All Fields] OR ("ocular"[All Fields] AND "infection"[All Fields]) OR "ocular infection"[All Fields])) OR "Eye/pathology"[Mesh]) OR ("eye pain"[MeSH Terms] OR ("eye"[All Fields] AND "pain"[All Fields]) OR "eye pain"[All Fields])) OR "Eye/injuries"[Mesh]) OR ("eye injuries"[MeSH Terms] OR ("eye"[All Fields] AND "injuries"[All Fields]) OR "eye injuries"[All Fields])) OR ("eye infections"[MeSH Terms] OR ("eye"[All Fields] AND "infections"[All Fields]) OR "eye infections"[All Fields])) OR ("eye diseases"[MeSH Terms] OR ("eye"[All Fields] AND "diseases"[All Fields]) OR "eye diseases"[All Fields]))
596533 Results

#4
Search: #1 AND #2 AND #3
933 Results

June 10, 2019

© 2020 Propst SL et al. *JAMA Network Open.*
